# Supplementary material for: Resolving the full spectrum of human genome variation using Linked-Reads
Source: Genome Res. 2019 Apr;29(4):635–45. doi: 10.1101/gr.234443.118 (PMC6442396; doi:10.1101/gr.234443.118)
Supplement: Supplemental Material [file supp_gr.234443.118_Supplemental_longranger-2.2.zip › longranger-2.2.tar.bz2!/lr2.2-public/lib/stan/cmdstan/stan/doc/api/html/math_2functions_2_phi_8hpp_source.html]

Stan: src/stan/math/functions/Phi.hpp Source File


|  |  |
| --- | --- |
|  | Stan  2.5.0  probability, sampling & optimization |


- Main Page
- Related Pages
- Namespaces
- Classes
- Files

- File List
- File Members

All Classes Namespaces Files Functions Variables Typedefs Enumerations Enumerator Friends Macros Pages

- src
- stan
- math
- functions

Phi.hpp

Go to the documentation of this file.

1 #ifndef STAN\_\_MATH\_\_FUNCTIONS\_\_PHI\_HPP

2 #define STAN\_\_MATH\_\_FUNCTIONS\_\_PHI\_HPP

3

4 #include <boost/math/tools/promotion.hpp>

5 #include <boost/math/special\_functions/erf.hpp>

6 #include <stan/math/constants.hpp>

7 #include <stan/math/error\_handling/check\_not\_nan.hpp>

8

9 namespace stan {

10  namespace math {

11

29  template <typename T>

30  inline typename boost::math::tools::promote\_args<T>::type

31  Phi(const T x) {

32  // overridden in fvar and var, so can hard-code boost versions

33  // here for scalars only

34  check\_not\_nan("Phi(%1%)", x, "x", static\_cast<double\*>(0));

35  if (x < -37.5)

36  return 0;

37  else if (x < -5.0)

38  return 0.5 \* boost::math::erfc(-INV\_SQRT\_2 \* x);

39  else if (x > 8.25)

40  return 1;

41  else

42  return 0.5 \* (1.0 + boost::math::erf(INV\_SQRT\_2 \* x));

43  }

44

45  }

46 }

47

48 #endif

check\_not\_nan.hpp

stan::agrad::erf

fvar< T > erf(const fvar< T > &x)

**Definition:** erf.hpp:17

stan::agrad::erfc

fvar< T > erfc(const fvar< T > &x)

**Definition:** erfc.hpp:17

stan::math::Phi

boost::math::tools::promote\_args< T >::type Phi(const T x)

The unit normal cumulative distribution function.

**Definition:** Phi.hpp:31

stan::math::INV\_SQRT\_2

const double INV\_SQRT\_2

The value of 1 over the square root of 2, .

**Definition:** constants.hpp:26

stan::math::check\_not\_nan

bool check\_not\_nan(const char \*function, const T\_y &y, const char \*name, T\_result \*result)

Checks if the variable y is nan.

**Definition:** check\_not\_nan.hpp:56

constants.hpp


---

[ Stan Home Page ]

*© 2011–2014,
Stan Development Team.*
